# Improving the use of research evidence in guideline development: 5. Group processes

**DOI:** 10.1186/1478-4505-4-17

**Published:** 2006-12-01

**Authors:** Atle Fretheim, Holger J Schünemann, Andrew D Oxman

**Affiliations:** 1 Norwegian Knowledge Centre for the Health Services, P.O. Box 7004, St. Olavs plass, N-0130 Oslo, Norway; 2 INFORMA, S.C. Epidemiologia, Istitituto Regina Elena, Via Elio Chianesi 53, 00144 Rome, Italy; 3 Norwegian Knowledge Centre for the Health Services, P.O. Box 7004, St. Olavs plass, N-0130 Oslo, Norway

## Abstract

**Background:**

The World Health Organization (WHO), like many other organisations around the world, has recognised the need to use more rigorous processes to ensure that health care recommendations are informed by the best available research evidence. This is the fifth of a series of 16 reviews that have been prepared as background for advice from the WHO Advisory Committee on Health Research to WHO on how to achieve this.

**Objective:**

In this review we address approaches to facilitate sound processes within groups that develop recommendations for health care.

**Methods:**

We searched PubMed and three databases of methodological studies for existing systematic reviews and relevant methodological research. We did not conduct systematic reviews ourselves. Our conclusions are based on the available evidence, consideration of what WHO and other organisations are doing and logical arguments.

**Key question and answer:**

What should WHO do to ensure appropriate group processes?

Various strategies can be adopted to ensure that the group processes in play when panels are developing recommendations are inclusive, so that all voices can be heard and all arguments given fair weight, including

• the use of formal consensus development methods, such at the Nominal Group Technique or the Delphi method

• the selection of a group leader who is qualified and responsible for facilitating an appropriate group process.

## Background

The World Health Organization (WHO), like many other organisations around the world, has recognised the need to use more rigorous processes to ensure that health care recommendations are informed by the best available research evidence. This is the fifth of a series of 16 reviews that have been prepared as background for advice from the WHO Advisory Committee on Health Research to WHO on how to achieve this.

A group that is convened to formulate recommendations will necessarily enter some sort of consensus development process. The panel-members will pass judgements on the available research evidence, consider various trade-offs (between expected benefits, harms and costs), and finally try to reach a consensus on what recommendation to make. The consensus may be reached through informal processes or more formal methods.

In this paper we address the following question: What should WHO do to ensure appropriate group processes? Questions related to group composition, integrating values and consumer involvement are addressed in other papers in this series [1,2].

## What is WHO doing now?

We are not aware of any examples of the use of formal consensus development methods by groups that have developed recommendations on behalf of WHO. In documents describing procedures for Expert Committees, it is stated that the meetings "shall normally be of private character" [3].

In our literature search we identified a paper describing the use of formal consensus development methods in modifying WHO's "Guidelines for the management of HIV/AIDS in adults and children" for use in Malawi and Barbados [4]. The method employed was the Nominal Group Technique.

## What are other organisations doing?

Several formal approaches for reaching consensus exist, and some organisations use these in the development of clinical practice guidelines. Three of the most common methods for reaching consensus are the Nominal Group Technique (NGT), the Delphi Method, and Consensus Conferences. A brief description of commonly used consensus development methods is found in Table 1. However, there is considerable variation in how these techniques are implemented in practice.

**Table 1:** Characteristics of various consensus development methods (from Murphy et al. [12])

Consensus development method	Mailed questionnaires	Private decisions elicited	Formal feedback of group choices	Face-to-face contact	Interaction structured	Aggregation method
Informal	No	No	No	Yes	No	Implicit
Delphi method	Yes	Yes	Yes	No	Yes	Explicit
NGT	No	Yes	Yes	Yes	Yes	Explicit
RAND version	Yes	Yes	Yes	Yes	Yes	Explicit
Consensus development conference	No	No	No	Yes	No	Implicit
Other methods						
Staticised group	No	Yes	No	No	-	Explicit
Social judgement analysis	No	Yes	Yes	Yes	No	Implicit
Structured discussion	No	No	No	Yes	Yes	Implicit

In a recent international survey of organisations that develop clinical practice guidelines or health technology assessments, approximately 42 % of the respondents reported using formal consensus development methods [5]. A smaller survey of prominent guideline developers reported that 7 of 18 programs used formal consensus methods to formulate recommendations [6].

With informal consensus development, a strategy is needed to ensure appropriate group processes. Typically responsibility for this is given to the group leader. Consequently, much weight is put on selecting the right person for this position. The National Institute for Health and Clinical Excellence (NICE) in the UK notes that the group leader "needs to allow sufficient time for all members to express their views without feeling intimidated or threatened and should check that **all **the members in the groups agree to endorse any recommendations" [7]. Furthermore, "The Chair should be selected as someone who is neutral and who has enough expertise in coordinating groups of health professionals and patients/carers so that the appointment is acceptable to all." [7].

## Methods

The methods used to prepare this review are described in the introduction to this series [8]. Briefly, the key questions addressed in this paper were vetted amongst the authors and the ACHR Subcommittee on the Use of Research Evidence (SURE). We searched PubMed and three databases of methodological literature (the Cochrane Methodology Register [9], the US National Guideline Clearinghouse [10] and the Guidelines International Network [11]) for existing systematic reviews and relevant methodological research that address these questions. We did not conduct systematic reviews ourselves. The answers to the questions are our conclusions based on the available evidence, consideration of what WHO and other organisations are doing and logical arguments.

In the literature search we used the term "consensus and process and method". We also checked the reference lists of key papers and contacted researchers in the field.

## Findings

### What should WHO do to ensure appropriate group processes?

One comprehensive review on consensus development methods was identified, and it provided most of the key findings for this report [12], together with an updated review that largely confirms the findings [13]. The review addresses three questions related to interaction within guideline development groups: 1) Does the choice of consensus development method influence the group's decision? 2) Does the setting for the group meetings affect the consensus decision? 3) Do the characteristics of a group facilitator affect the consensus decision?

Various measurements of decision quality were used as outcome measures in the comparative studies that were included in the review. For instance, comparison with "gold standard", such as asking the groups to reach an agreement on "questions that have correct answers which the participants do not know with any precision," e.g. "What is the diameter of Jupiter?". For ranking tasks (e.g. "to rank items in terms of their value for survival on the moon"), the group decision "can be compared with rankings by experts." The applicability of these types of studies for processes taking place within guideline development groups is not obvious.

For choice of consensus development methods, the reviewers identified 16 studies comparing NGT with informal methods, 11 comparing the Delphi method with informal methods, and seven studies comparing NGT and Delphi. Interpreting the results is not straight-forward since "the studies also differ in the particular way they operationalise the method used". The reviewers did not find any comparative studies involving consensus development conferences. Their summary conclusion was that "Formal methods generally perform as well or better than informal methods, but it is difficult to tell which of the formal methods is best."

With regards to the settings for group meetings, the reviewers concluded that "There is little research which actually looks at this question. However, of the many factors which can influence decision-making, except for extreme environments, the environment is likely to have only a marginal impact."

Concerning characteristics of a group facilitator, the research base is difficult to interpret as "the models of leadership used are often not directly transferable to facilitation". Although there is "very little work that looks at the effects of facilitation on group decision-making", the reviewers believe that "it is likely that this key role will influence group decision-making."

We identified one additional study that compared informal consensus with a formal consensus method ("the appropriateness method") for developing clinical practice guidelines on the management of low-back pain [14]. The investigators found that guideline statements resulting from the two approaches were "qualitatively similar", however the formal method produced statements that in some instances were "more clinically specific".

## Discussion

The idea of bringing people together to develop recommendations is based on the understanding that they all have something to contribute. Thus, it is essential to secure that all participants can be heard and have the opportunity of influencing the outcome of the process. This is a common understanding among groups that develop guidelines, and many have therefore adopted specific strategies to ensure appropriate group processes.

Given the costs of group meetings, different languages and cultural differences, it is especially important for WHO to ensure that all of the invited members contribute fully to the development of recommendations. Transparency is important to ensure that groups know and adhere to the methods that they are supposed to be use. For instance, the group may report that they base their recommendations on research evidence, while they in reality reach their conclusions on a different basis. A qualitative study of decision-making processes within drug-selection committees in hospitals in the UK, for example, found that many decisions were not based on research findings, despite being reported as if they were: "reports of decisions...are written so as to account for the decision in terms of scientific rationality...rather than the local rationality that was actually employed" [15].

The research base to inform the choice of strategy to ensure appropriate group processes is limited, however in addition to logical arguments there is also some empirical evidence in support of using formal consensus development methods rather than relying only on informal processes. Having a group leader that facilitates the group process is likely essential. Conflicts may arise within groups and the leader of the group will have an important role in trying to manage these. Dealing with conflict is usually a difficult task, and WHO should consider establishing routines to support groups in managing these.

A weakness of our review was the literature search was limited to PubMed and three databases of methodological literature and did not include additional searches in the social science literature.

## Further work

In general, there is need for research to learn more about the relative merits of various methods for facilitating sound group processes. Head to head comparisons of different consensus development methods within groups that develop recommendations for health care should be done, since most research so far has taken place in very different settings. Also, research is needed to identify the most critical selection criteria and processes for selecting a chairperson for groups developing recommendations.

## Competing interests

AF and ADO work for the Norwegian Knowledge Centre forthe Health Services, an agency funded by the Norwegian government that produces systematic reviews and health technology assessments. All three authors are contributors to the Cochrane Collaboration. ADO and HJS are members of the GRADE Working Group. HJS is documents editor and chair of the documents development and implementation committee for the American Thoracic Society and senior editor of the American College of Chest Physicians' Antithrombotic and Thrombolytic Therapy Guidelines.

## Authors' contributions

AF prepared the first draft of this review. HJS and ADO contributed to drafting and revising it.
